# Evaluation of an Automated Ultrafiltration System for Concentrating a Range of Viruses from Saline Waters

**DOI:** 10.1007/s12560-024-09602-6

**Published:** 2024-06-29

**Authors:** Simran Singh, Tiong Gim Aw, Joan B. Rose

**Affiliations:** 1 Department of Fisheries and Wildlife, Michigan State University, East Lansing, MI USA; 2 Department of Environmental Health Sciences, School of Public Health and Tropical Medicine, Tulane University, 1440 Canal Street, Suite 2100, New Orleans, LA 70112 USA

**Keywords:** Ultrafiltration, Automated bio-concentrator, Marine water, Viruses, Droplet digital PCR, Environmental monitoring

## Abstract

Pathogenic viruses in environmental water are usually present in levels too low for direct detection and thus, a concentration step is often required to increase the analytical sensitivity. The objective of this study was to evaluate an automated filtration device, the Innovaprep Concentrating Pipette Select (CP Select) for the rapid concentration of viruses in saline water samples, while considering duration of process and ease of use. Four bacteriophages (MS2, P22, Phi6, and PhiX174) and three animal viruses (adenovirus, coronavirus OC43, and canine distemper virus) were seeded in artificial seawater, aquarium water, and bay water samples, and processed using the CP Select. The recovery efficiencies of viruses were determined either using a plaque assay or droplet digital PCR (ddPCR). Using plaque assays, the average recovery efficiencies for bacteriophages ranged from 4.84 ± 3.8% to 82.73 ± 27.3%, with highest recovery for P22 phage. The average recovery efficiencies for the CP Select were 39.31 ± 26.6% for adenovirus, 19.04 ± 11.6% for coronavirus OC43, and 19.84 ± 13.6% for canine distemper virus, as determined by ddPCR. Overall, viral genome composition, not the size of the virus, affected the recovery efficiencies for the CP Select. The small sample volume size used for the ultrafilter pipette of the system hinders the use of this method as a primary concentration step for viruses in marine waters. However, the ease of use and rapid processing time of the CP Select are especially beneficial when rapid detection of viruses in highly contaminated water, such as wastewater or sewage-polluted surface water, is needed.

**Graphical abstract:**

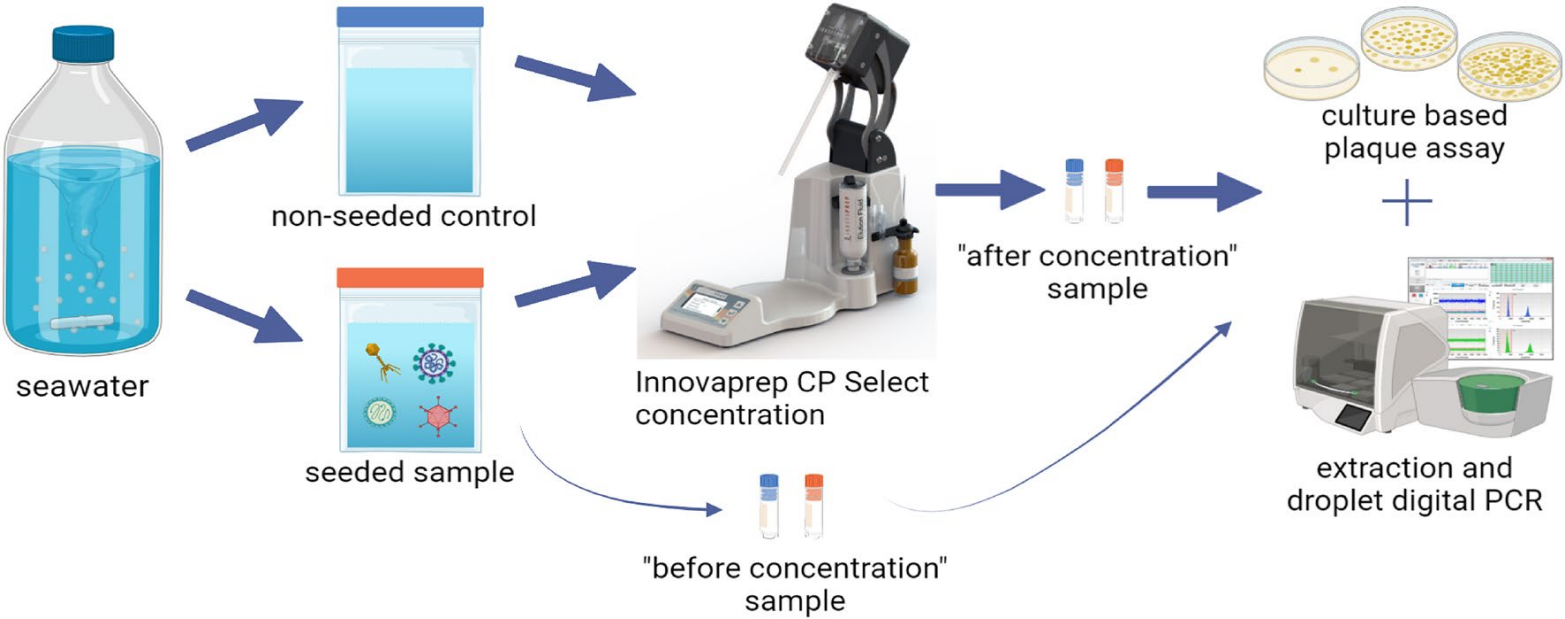

## Introduction

The ability to rapidly detect viruses in the environment can prevent disease transmission between animals or to humans. However, the detection of viruses in environmental waters constitutes special challenges, and multi-stage concentration procedures of water samples are typically needed. Continued development and evaluation of methods for concentrating viruses in environmental waters is of critical importance. In general, analysis of water for viruses requires concentration steps because the ambient quantity of pathogenic viruses in environmental waters is usually less than an assay’s limit of detection.

An ideal concentration method should be technically simple, rapid, inexpensive, and be able to provide high virus recovery rate with a small volume of final concentrate and concentrate a wide range of virus groups simultaneously (Wyn-Jones & Sellwood, 2001). To date, there is no consensus on the most efficient approach for concentrating all viruses in water samples. The first stage of concentration normally comprises a technique such as an adsorption-elution method which uses electronegative or electropositive filters or ultrafiltration which reduces the initial volume of water sample to between 100 and 500 mL (Cashdollar & Wymer, 2013; Hill et al., 2005). The secondary concentration such as organic flocculation is needed for reconcentrating viruses to a sample volume of less than 10 mL. Water matrix characteristics such as turbidity, pH, salinity, organic matter could influence the efficacy of concentration methods and the recovery of viruses (Gibbons et al., 2010). Most of the current concentration methods such as skimmed-milk flocculation, polyethylene glycol (PEG) precipitation, ultracentrifugation, virus adsorption-elution (VIRADEL) using NanoCeram filters or beef extract-celite, are time-consuming, not automated, and difficult to deploy in the field (Wyn-Jones & Sellwood, 2001; Falman et al., 2019; Forés et al., 2021; Gonzalez et al., 2020; Rusiñol et al., 2020).

Recently, a new technology, Concentrating Pipette Select (CP Select; InnovaPrep, Drexel, MO, USA), has been developed for sampling and processing water for pathogen detection. The CP Select is an automated and compact filtration device. For viruses, the one-pass method works by filtration through high-flow single-use pipette tips to remove viruses from the sample matrix. Once the viruses are captured, the instant and automated wet foam elution process recovers the viruses into an average 200 µL volume of clean buffer for analysis. The CP Select has been mainly used for detecting viruses such as MS2 phages, noroviruses, and coronaviruses (SARS-CoV-2) in untreated wastewater (Ahmed et al., 2021; Forés et al., 2021; Gonzalez et al., 2020; Juel et al., 2021; Lee et al., 2021; Lu et al., 2022; McMinn et al., 2021; Rusiñol et al., 2020). The CP Select concentration method has not been evaluated for use with surface water such as saline water to recover a range of viruses.

The spread of viruses to marine mammals in enclosed habitats is of concern for animal health (Suttle, 2005; Jo et al., 2018). Animals that exist in enclosures and aquaria may be particularly susceptible to disease as these are sites where contact between humans and marine mammals occurs frequently which can lead to disease transmission, such as transferring localized skin infections from animals to humans or vice versa (Suttle, 2005; Waltzek et al., 2012; Wellehan & Cortes-Hinojosa, 2019). These infections have occurred in many recreational industries (whale-watching tours, oceanaria, and ‘swimming with dolphins’ programs) as well as during research, and interactions with rehabilitators, trainers, veterinarians, and volunteers (Wei et al., 2020; Waltzek et al., 2012). Therefore, it is important to develop a non-invasive, early surveillance method for viral pathogens in marine environments to protect both animals and humans. Thus, the objective of this study was to evaluate the CP Select as an automated approach to recover seven viruses from saline water samples for environmental surveillance.

## Material and Methods

### Sources of Water Samples

Artificial seawater samples were prepared by adding approximately 40 g of Instant Ocean salts (Instant Ocean, Blacksburg, VA, USA) to 2 L of sterile ultrapure water. The conductivity, pH, and turbidity for artificial seawater samples in this study were 29 to 40.0 ± 8 mS/cm, 8.00 ± 0.05, and 7.49 ± 0.05 NTU, respectively. Aquarium water sample (conductivity, 48 mS/cm; pH, 7.5; turbidity, 0.1 NTU) and San Diego Bay water sample (conductivity, 48.8 mS/cm; pH, 7.7; turbidity, 0.2 NTU) were also used.

### Virus Cultures Preparation

Four bacteriophage and three animal viruses with different genome types and sizes of virus particle were used (Table 1). Phages MS2 (American Type Culture Collection, ATCC 15597-B1), PhiX174 (ATCC 13706-B1) and P22 were propagated on *Escherichia coli* (*E. coli*) Famp (ATCC 700891), *E. coli* CN-13 (ATCC 700609), and *Salmonella typhimurium* LT2 (HER 1023; the Félix d’Hérelle Reference Center for bacterial viruses, Université Laval, Québec, Canada), respectively, and enumerated using the double agar overlay plaque assay following U.S. EPA method 1602 (EPA, 2001). To propagate phage Phi6, *Pseudomonas syringae* was grown in King’s B medium at 20 °C for 6 h. Phi6 phages were added to the host and incubated under the same conditions for 16–18 h. All the bacteriophage culture stocks were filtered using 0.22 µm polyethersulfone (PES) membrane (MilliporeSigma, St. Louis, MO, USA) to remove host bacteria and titered using plaque assay. Virus titers of approximately 10^6^ to 10^8^ plaque forming unit (PFU) per mL were routinely obtained.

**Table 1:** Characteristics of bacteriophages and animal viruses used in this study

Virus	Virus family	Baltimore classification	Diameter (nm)	Enveloped or naked
MS2	*Leviviridae*	IV ((+) ssRNA^a^)	24–27	Naked
P22	*Podoviridae*	I (dsDNA^b^)	52–60	Naked
Phi6	*Cystoviridae*	III (dsRNA^c^)	80–100	Enveloped
PhiX174	*Microviridae*	II (ssDNA^d^)	23–27	Naked
Adenovirus type 10	*Adenoviridae*	I (dsDNA)	70–100	Naked
Coronavirus OC43	*Coronaviridae*	IV ((+) ssRNA)	80–120	Enveloped
Canine distemper virus (CDV)	*Paramyxoviridae*	V ((−) ssRNA)	150–300	Enveloped

^a^ssRNA single-stranded ribonucleic acid

^b^dsDNA double-stranded deoxyribonucleic acid

^c^dsRNA double-stranded ribonucleic acid

^d^ssDNA single-stranded deoxyribonucleic acid

(+) denotes positive-sense and (−) denotes negative-sense RNA

Human adenovirus 10 (ATCC VR1504) was grown using the A549 cell line (ATCC CCL-185). Briefly, virus suspension was inoculated onto monolayer of recently passaged A549. The cells were incubated with Dulbecco’s modified Eagle medium (DMEM) containing 2% fetal bovine serum at 35.5 °C. Following the development of 80% cytopathic effect, the tissue culture flasks containing cells were frozen at −80 °C and thawed three times. The resulting suspension was centrifuged at 10,000 × g for 20 min, and the supernatant was filtered through 0.22 µm PES filter. The same protocol was used to propagate coronavirus OC43 (ATCC VR1558) using the HRT-18G cell line (ATCC CCL-244). The canine distemper virus (CDV; ATCC VR-1587) was propagated using the Vero cell line (ATCC CCL-81) (Ammerman et al., 2008).

### Virus Concentration Steps Using the CP Select

For each experiment, 1 mL of the virus stock between 10^6^ and 10^8^ PFU (depending on the virus) was spiked into 500 mL of water samples. After mixing, 5 mL of water sample was removed for use in determining the seeded virus level prior to the concentration steps. The remaining water sample was transferred into a Whirl–Pak bag (Whirl–Pak, Fort Atkinson, WI, USA), and then processed using the CP Select with ultrafilter pipette tips (Item no. CC08003) according to the manufacturer’s recommended settings: 100% pump rate; valve open 800 ms; valve closed 100 ms; pulse 2; foam factor 10; flow start 3.0 s; flow end 0.2 s; extended delay 3 s and extended pump delay 1 s (Birkenholz, 2020). The pipette was eluted using the automated wet foam elution process with FluidPrep Elution Fluid Can- Tris (InnovaPrep, Drexel, MO, USA). The range of elution volumes for one pipette was between 0.65 and 1.9 mL. In this study, virus concentration factors between 60- and 200-fold were achieved. Viruses in concentrated water samples were enumerated using plaque assays and/or molecular method. All the experiments were performed in at least triplicate.

### Nucleic Acid Extraction and ddPCR Assays

Viral RNA and DNA were extracted using separate extraction kits. RNA was extracted from concentrates using the QIAamp Viral RNA Mini kit (QIAGEN, Hilden, Germany) according to the manufacturer’s protocol. In this study, a total of 140 µL of concentrate was used for RNA extraction resulting in a final elution volume of 80 µL. DNA was extracted using QIAamp DNA Mini kit (QIAGEN, Hilden, Germany) according to the manufacturer’s protocol. A total of 200 µL of concentrate was used for DNA extraction resulting in a final elution volume of 200 µL.

Droplet digital PCR (ddPCR) was also used to quantify the viral nucleic acid to determine the recovery efficiencies of the concentration method. All the primers and probes used in this study are listed in Table 2. The primers and probes were adapted from quantitative PCR (qPCR) protocols which were optimized for ddPCR. For RNA viruses, ddPCR was performed using Bio-Rad’s one-step RT-ddPCR Advanced Kit with a QX200 ddPCR system (Bio-Rad, CA, USA). Each reaction contained a final concentration of 5.5 µL of 1 × Supermix (Bio-Rad, CA, USA), 2.2 µL of reverse transcriptase (RT), 1.1 µL of 15 mM DTT, 3.3 µL of primer–probe mixture (0.9 µM of each primer and 0.25 µM of probe), 4.4 µL of molecular-grade RNAse-free water, and 5.5 µL of template RNA for a final reaction volume of 22 µL. For DNA viruses, each ddPCR reaction contained a final concentration of 11 µL of 2 × Supermix for probes, no dUTP (Bio-Rad, CA, USA), 2 µL of each 10 µM primer, 0.6 µL of 10 µM probe, 0.9 µL of molecular-grade RNAse-free water, and 5.5 µL of template DNA for a final reaction volume of 22 µL.

**Table 2:** Primers and probes used for ddPCR

Virus	Sequence (5′–3′)	PCR reaction profile	Reference
MS2	TGCTCGCGGATACCCG	25 °C, 3 min; 47 °C, 60 min; 95 °C, 10 min; 40 cycles of 95 °C, 30 s; 55 °C, 1 min with ramp rate of 2 °C s-1; 98 °C, 10 min; held at 4 °C	Trojnar et al. (2020)
	AACTTGCGTTCTCGAGCGAT		
	[FAM]ACCTCGGGTTTCCGTCTTGCTCGT[BHQ1]		
Phi6	TGGCGGCGGTCAAGAGC	25 °C, 3 min; 50 °C, 60 min; 95 °C, 10 min; 40 cycles of 95 °C, 30 s; 60 °C, 1 min with ramp rate of 2 °C s-1; 98 °C, 10 min; held at 4 °C	Flood et al. (2021)
	GGATGATTCTCCAGAAGCTGCTG		
	[FAM]CGGTCGTCGCAGGTCTGACACTCGC[BHQ1]		
Coronavirus OC43	CGATGAGGCTATTCCGACTAGGT	25 °C, 3 min; 50 °C, 1 h; 95 °C, 10 min; 40 cycles of 95 °C, 30 s; 55 °C, 1 min with ramp rate of 2 °C s-1; 98 °C, 10 min; held at 4 °C	Pecson et al. (2021)
	CCTTCCTGAGCCTTCAATATAGTAACC		
	[FAM]TCCGCCTGGCACGGTACTCCCT[BHQ1]		
Adenovirus	GGACGCCTCGGAGTACCTGAG	95 °C, 10 min; 39 cycles of 94 °C, 30 s; 55 °C, 1 min with ramp rate of 2 °C s-1; 98 °C, 10 min; held at 4 °C	Jothikumar et al. (2005)
	ACIGTGGGGTTTCTGAACTTGTT		
	[FAM]CTGGTGCAGTTCGCCCGTGCCA[BHQ1]		
CDV	CTGTCRGTAATCGAGRATTCGA	25 °C, 3 min; 42 °C, 1 h; 95 °C, 10 min; 40 cycles of 95 °C, 30 s; 55 °C, 1 min with ramp rate of 2 °C s-1; 98 °C, 10 min; then held at 4 °C	Halecker et al. (2021)
	GCCGAAAGAATATCCCCAGTTAG		
	[FAM]ATCTTCGCCAGARTCYTCAGTGCT[BHQ1]		

Droplet generation was performed by microfluidic mixing of 20 µL of each reaction mixture with 70 µL of droplet generation oil in a droplet generator (Bio-Rad, CA, USA). The final volume transferred into the 96 well plate was 40 µL of reaction mixture–oil emulsions. The plate was sealed and then placed into a C1000 96-deep well thermocycler (Bio-Rad, CA, USA) for PCR. Following PCR thermocycling, the plate was transferred to a QX200 Droplet Reader (Bio-Rad, CA, USA) to determine the gene concentration through the detection of positive droplets containing each gene target by spectrophotometric detection of the fluorescent probe signal. Quality controls were run with every plate including positive and non-template controls, extraction controls, and processing blanks for each experiment.

### Statistical Analysis

Recovery efficiency for the plaque assay was calculated by dividing the “after concentration” PFU by the “before concentration” PFU and then multiplied by 100. Similarly, for ddPCR assay, recovery efficiency was calculated by dividing the total gene copies (GC) per 500 mL “after concentration” by the “before concentration” then multiplied by 100 (Flood et al., 2021).

$$\frac{{{\text{After Concentrate}}{\mkern 1mu} \left( {{\text{PFU or}}{\mkern 1mu} \frac{{{\text{GC}}}}{{{\text{mL}}}}} \right)}}{{{\text{Before Concentrate}}{\mkern 1mu} \left( {{\text{PFU or}}{\mkern 1mu} \frac{{{\text{GC}}}}{{{\text{mL}}}}} \right)}} \times 100 = {\text{Recovery Efficiency}}{\mkern 1mu} \%$$

All MS2, Phi6, Adenovirus, OC43, and CDV data were converted from GC per reaction to GC per volume of sample. Negative samples were accepted if less than three droplets were above the set threshold. PCR inhibition was accounted for by diluting all sample extractions to be within the detectable range of the ddPCR system and calculations were adjusted accordingly. The detection limit for RNA samples was 342.85 GC per 500 mL and DNA samples was 600 GC per 500 mL, when accepted droplets were 20,000.

$${\text{Virus}}\,{\text{GC}}\,{\text{per}}\,500\,{\text{mL}} = \frac{{\left( {\frac{{{\text{GC}}\,{\text{per}}\,{\text{rxn}}}}{V_r }} \right) \times V_e \times \left( {\frac{V_f }{{V_c }}} \right)}}{V_i } \times 500$$

where *V*_*i*_ = initial volume of sample before concentration (mL), *V*_f_ = final volume of sample after concentration (mL), *V*_r_ = volume of RNA template used per PCR reaction (µL), *V*_e_ = final volume for DNA or RNA eluted from extraction (µL), *V*_c_ = volume of concentrated sample used for extraction (mL) (Flood et al., 2021).

Statistics and data visualization were executed using Graphpad Prism 9.5.1 (Graphpad Software, CA, USA). The significance of experimental variables for bacteriophage using plaque assay was determined using ordinary one-way analysis of variance (ANOVA). For bacteriophages tested using ddPCR, Phi6, and MS2, an unpaired t test with Welch’s correction was used as well as F test to compare variances. To investigate the effect of sample matrix type on recovery of animal viruses using ddPCR, the two-factor ANOVA test was used with significance level p < 0.05.

## Results and Discussion

### The Recovery of Viruses in Saline Waters Using the CP Select

**Fig. 1:**
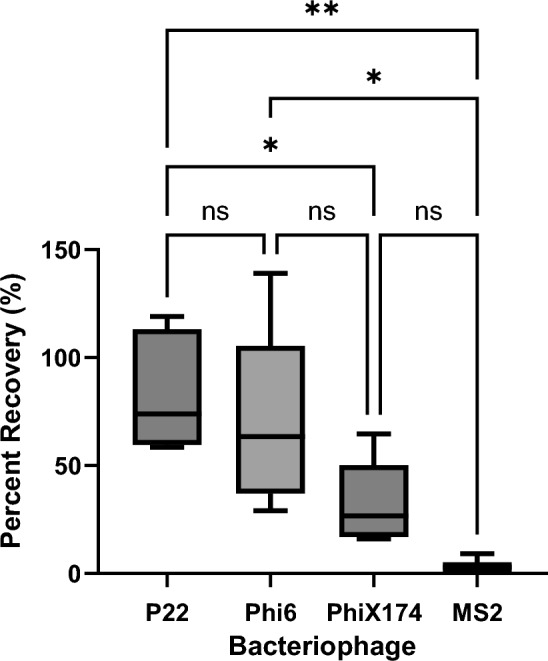
Comparison of the recovery of culturable bacteriophage P22, MS2, PhiX174, and Phi6 in artificial seawater using the CP Select. Bacteriophages before and after the concentration steps were enumerated using plaque assays. Multiple comparisons one-way ANOVA results shown above: *ns* not significant, *p value < 0.05, **p value < 0.005

Using plaque assays, the average recovery efficiencies of the CP Select for bacteriophage P22, Phi6, PhiX174, and MS2 in artificial seawater samples were 82.73 ± 27.3%, 71.48 ± 42.5%, 32.77 ± 19.3%, and 4.84 ± 3.8%, respectively (Fig. 1). The ordinary one-way ANOVA showed statistically significant difference (*F* = 6.473, DFn = 3, DFd = 17, *p* = 0.0040).

At present, molecular techniques have been widely used to detect viruses in environmental water samples. Therefore, in this study, the performance of the CP Select in recovering viral particles using nucleic acid detection was also assessed. Using ddPCR assays, the average recovery efficiencies for MS2 and Phi6 RNA were 7.82 ± 8.1% and 34.72 ± 19.7%, respectively, and these were statistically significantly different (*p* = 0.0233).

**Fig. 2:**
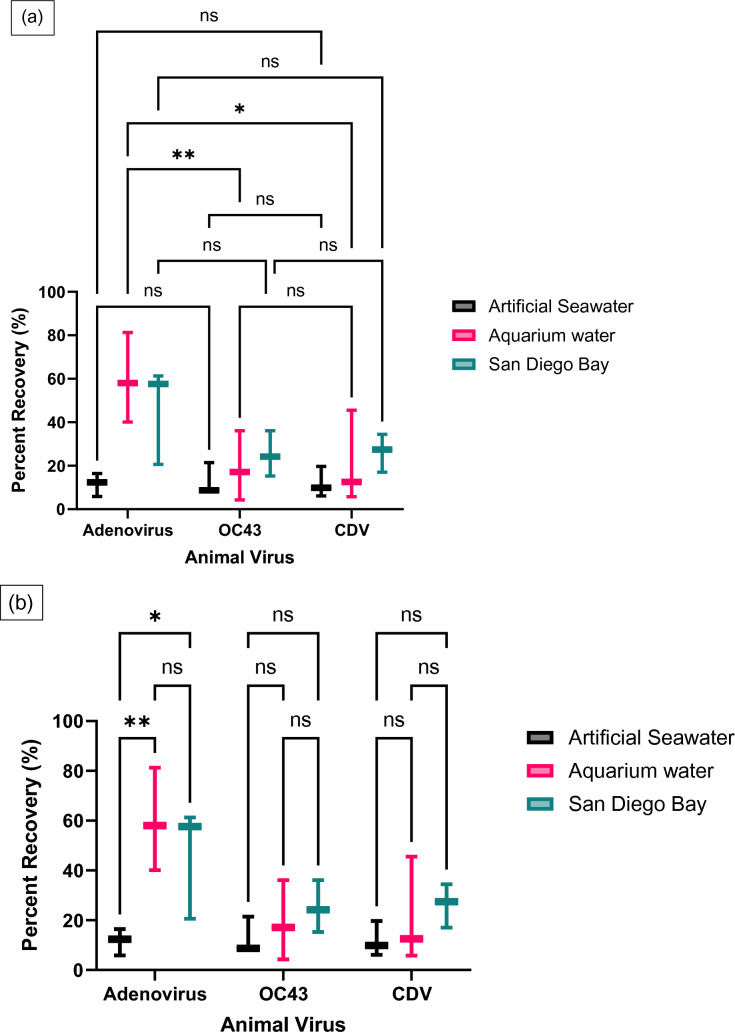
The recovery efficiencies of adenovirus, coronavirus OC43, and CDV in different water samples using the CP Select. Virus concentrations before and after the concentration steps were determined using ddPCR. **a** Comparison of virus recoveries within a matrix. **b** Comparison of a virus recovery between matrixes. Multiple comparisons two-way ANOVA results shown above: *ns* not significant, *p value < 0.05, **p value < 0.005

The average recovery efficiencies of adenovirus in artificial seawater, aquarium water, and bay water using the CP Select were 11.58 ± 5.3%, 59.81 ± 20.6%, and 46.53 ± 22.5%, respectively (Fig. 2). Regardless of the water sample type, the overall average recovery efficiency of adenovirus was 39.31 ± 26.6%. The recovery efficiencies for coronavirus OC43 and CDV were comparable (Fig. 2). The artificial seawater tests resulted in 12.75 ± 7.6% recovery for OC43 and 11.91 ± 7.0% for CDV. The CP Select had an average percentage recovery of 19.16 ± 16.0% and 21.28 ± 21.3% for OC43 and CDV in aquarium water, respectively. When viruses were seeded into the bay water, the average percentage recovery of 25.22 ± 10.4% for OC43 and 26.32 ± 8.8% for CDV was observed. Overall, average recovery efficiencies of the CP Select were 19.04 ± 11.6% for OC43 and 19.84 ± 13.6% for CDV in waters. The recoveries of viruses were significantly different from each other in the same water matrix, e.g., in aquarium water, adenovirus was recovered differently than OC43 and CDV (*F* = 5.444, DFn = 2, DFd = 18, *p* = 0.0142) (Fig. 2a). In addition, the matrix had a statistically significant impact on the recovery of adenoviruses (*F* = 6.064, DFn = 2, DFd = 18, *p* = 0.0097) (Fig. 2b).

### The Effect of Salinity and Virus Properties on Virus Recovery

We also examined the effect of salinity in recovering adenovirus and Phi6 phage from artificial seawater samples using the CP Select and ddPCR. The average recovery efficiencies for adenovirus and Phi6 were 18.12 ± 6.9% (*n* = 3) and 22.58 ± 9.8% (*n* = 3), respectively, for water samples with a salinity of 29.0 mS/cm. For water samples with higher salinity (49.0 mS/cm), average recovery efficiencies of adenovirus and Phi6 were 2.82 ± 0.2% (*n* = 3) and 4.73 ± 1.0% (*n* = 3), respectively. However, these differences between the viruses were not statistically significant (t = 3.8849, *df* = 2, *p* = 0.0612 for adenovirus, and t = 3.129, *df* = 2, *p* = 0.0863 for Phi6) using a Welch’s *T* test.

The two viruses with the highest average recoveries using ddPCR were phage Phi6 and adenovirus. Phage P22 had the highest average recovery for the CP Select when a plaque assay was used. Adenovirus, Phi6, and P22 all have double-stranded nucleic acid. In this study, the DNA viruses were found to have higher recoveries than the RNA viruses. The size range of the DNA viruses used in this study is between 23 and 100 nm while the RNA viruses are 24–300 nm in diameter. Since the ultrafiltration technique was used, we hypothesized that Phi6 (80–100 nm in diameter) and morbillivirus CDV (150–300 nm diameter) would have the highest percent recovery compared to all the other viruses. Larsen et al. (2023) compared varying ultrafilters and examined the recovery of dsDNA viruses; they found that recovery was highly dependent on the sample matrix and filter type (Larsen et al., 2023). However, this study demonstrated that the type of viral genome seemed more influential than the size of the virus in evaluating the performance of virus concentration methods. A study comparing a variety of viruses also reported that viral genetic material, structure, along with similar environmental factors could influence viral recovery (Farkas et al., 2022).

In this study, the highest percent recovery for P22 phage (52–60 nm diameter) could be due to high persistence of the P22 at a wide range of pH (4–8), and the pH of water matrices in this study ranged from 6.5 to 8.0. Coliphage MS2 had the lowest percent recovery among all the viruses tested in this study. This could be due to its small size, capsid thinness, or instability of the MS2 under high pH or salinity. Although seawater contains sodium chloride (NaCl), which could improve the recovery of small single-stranded RNA viruses using adsorption-elution methods. However, extremely high salt can adversely affect virus recovery using ultrafiltration technique (Ikner et al., 2012; Lukasik et al., 2000). Lukasik et al. (2000) found magnesium chloride increased virus adsorption by possibly strengthening hydrophobic interactions. The type of salt appeared to have different influences on virus adsorption to negatively and positively charged filters, with aluminum chloride having the most interference over sodium chloride and magnesium chloride (Lukasik et al., 2000). Further study is needed to evaluate the effect of salinity in concentrating viruses in saline water using ultrafiltration technique and the appropriateness of MS2 as a surrogate for animal viruses.

### Advantages and Disadvantages of the Concentration Method

There is no one concentration method that is effective at recovering all types of viruses which is partly due to their diversity in size and structure (Petrova et al., 2020). Advantages of the CP Select system include rapid sample processing, automatic and compact device, which allows for field applications. The filtration process does not require preconditioning of the filter or sample and multiple sample transfer steps to avoid sample cross-contamination (Boujnouni et al., 2022). In this study, the flow rates of the CP Select for filtering saline water were on average 17 mL per min and the concentration process took less than an hour. The CP Select is fairly user friendly and thus it is easy to train a new user. However, this concentration method also has several limitations. The ultrafilter pipette tips clog easily, which can be unfavorable when testing environmental samples where the concentrations of naturally occurring viruses are extremely low and turbidity is higher. In this study, the turbidity of water samples was low, so the clogging was likely due to high salinity of the samples, creating salt bridges that caused the viruses to coagulate and attach to the filter then not release during the elution (Heffron & Mayer, 2016; Lukasik et al., 2000). Taligrot et al. (2022) also experienced ultrafiltration filter membrane fouling when concentrating viruses in seawater but no fouling when concentrating fresh mineral water. Ultrafiltration uses size exclusion as the concentration mechanism, but viruses may become adsorbed to the filter’s membrane via van der Waals interactive forces and/or hydrophobic bonding (Ikner et al., 2012). This could be an explanation of viral loss during concentration steps in this study. In this study, filtering the water until the pipette tip fouls and eluting the pipette once resulted in the highest recoveries for seeded experiments. One pipette and one elution per sample is suggested because increasing number of pipettes used or elutions per pipette did not increase virus recovery but instead adds to the cost of consumables.

There are nine key published studies that have evaluated the CP Select for concentrating viruses in environmental samples (Table 3). All the studies used wastewater or contaminated river water. The initial sample volumes ranged between 40 and 500 mL. These previous studies showed that the CP Select has a wide range of recovery efficiencies for non-enveloped viruses such as poliovirus and coliphage MS2 (0.32–51%) in wastewater (Table 3). In this study, a lower recovery efficiency was also observed for MS2 in artificial seawater. For enveloped viruses such as coronaviruses in wastewater, recovery efficiencies of the CP Select ranging from 5.5 to 65% were reported in previous studies (Table 3). A previous study also suggests that the CP Select is more efficient at concentrating coronaviruses from wastewater as compared to the membrane adsorption/elution technique (Juel et al., 2021). Farkas et al. (2022) compared 11 viruses and 5 concentration methods in wastewater. The median recovery was 5.1% for all viruses using the CP Select (Farkas et al., 2022).

**Table 3:** Previously published studies on the CP Select for concentrating viruses in wastewater

Virus	Sample type	Sample volume (mL)	Average recovery %	References
Poliovirus type 1	Wastewater influent	100	0.32	Falman et al. (2019)
MS2 phage	Wastewater	80	51	Rusiñol et al. (2020)
Bovine coronavirus	Wastewater influent	100	5.5 ± 2.1	Gonzalez et al. (2020)
Bovine respiratory syncytial virus			7.6 ± 3.0	
Coronavirus OC43	Primary treated wastewater	100	22 ± 4	McMinn et al. (2021)
MS2 phage	Wastewater influent	100	27.72 ± 24.46	Forés et al. (2021)
Murine herpesvirus			7.51 ± 6.14	
Bovine coronavirus	Wastewater	40–100	36.81	Juel et al. (2021)
Gamma-irradiated SARS-CoV-2	Wastewater influent	50	65 ± 23.6	Ahmed et al. (2021)
Norovirus	Sewage-impacted river water	500	4.5	Lee et al. (2021)
Coronavirus OC43	Wastewater	100	15.5 ± 7.6	Lu et al. (2022)

In conclusion, the performance of a compact and automated bio-concentration device for the recovery of viruses in saline water samples was assessed. This device works well in saline waters and allows for concentrating a variety of viruses in a rapid sample processing turnaround time. However, it may not be used as a primary concentration method for viruses in a large volume of natural environmental waters due to a low detection limit for viruses. This approach could be used to concentrate viruses in highly contaminated environmental samples such as wastewater or sewage-polluted surface water, or as a secondary concentration procedure.
